# Mechanisms Linking Body Weight to Early Puberty in Children

**DOI:** 10.3390/children13101341

**Published:** 2026-10-02

**Authors:** Nicolas C. Nicolaides, Meropi Toumba, Aliaksei Tsishkavets, Numan Sakhi, Nicos Skordis

**Affiliations:** 1 Molecular and Translational Pediatric Endocrinology Laboratory, Cyprus Cancer Research Institute, Nicosia 1678, Cyprus; nicolaides.nicolas@ucy.ac.cy; 2 Medical School, University of Cyprus, Nicosia 1678, Cyprus; 3 Pediatric Endocrine Clinic, American Medical Center, Nicosia 2047, Cyprus; meropi.toumba@doctors.org.cy; 4 School of Medicine, University of Nicosia, Nicosia 2417, Cyprus; tsishkavets.a@live.unic.ac.cy (A.T.);; 5 Division of Paediatric Endocrinology, Paedi Center for Specialized Paediatrics, Nicosia 2025, Cyprus

**Keywords:** childhood obesity, leptin, insulin, sirtuins, kisspeptin, endocrine disrupting chemicals, pubertal onset, early puberty

## Abstract

**Highlights:**

**What are the main findings?**

- Childhood overweight and obesity are associated with altered pubertal timing, particularly earlier pubertal development in girls. Adipose tissue acts as a true endocrine organ, releasing multiple signaling molecules that can modulate the onset of puberty.
- The mechanisms linking adipose tissue excess to changes in pubertal timing are complex and remain incompletely established. Leptin provides a key permissive cue that links energy sufficiency to activation of the hypothalamic–pituitary–gonadal axis. Insulin, particularly in states of hyperinsulinemia, may further shape pubertal timing by influencing hypothalamic kisspeptin pathways as well as ovarian and adrenal androgen production. Environmental endocrine-disrupting chemicals—notably bisphenol A and phthalates—may also serve as intermediaries linking adiposity with pubertal development. Additional proposed mechanisms which need further investigation include sirtuin (SIRT1) dependent epigenetic regulation of Kiss1, interactions within the ghrelin–LEAP2 system, and contributions from the gut microbiota.

**What are the implications of the main findings?**

- Higher childhood BMI in girls is consistently associated with earlier thelarche, earlier menarche, and increased rates of earlier puberty. Adiposity-driven endocrine changes can predispose adolescents to irregular cycles, and metabolic–reproductive syndromes with increased risk of long-term reproductive and metabolic consequences with increased risks of infertility and metabolic syndrome.
- Further longitudinal and mechanistic research is needed to clarify causality and sex-specific effects. Research that integrates body composition, metabolic and hormonal measurements with genetic, epigenetic, microbiome, and environmental exposure data may provide evidence for understanding the relationship between obesity, pubertal timing, and subsequent cardiometabolic risk.

**Abstract:**

Childhood overweight and obesity have been associated with earlier pubertal onset most commonly in girls than boys. This review aims to summarize proposed mechanisms involved in early puberty amongst children with increased weight and possibly help early detection and reduce any long-term related risks associated with precocious puberty in overweight and obese children. Adipose tissue functions as an endocrine organ and produces several signaling molecules that may influence puberty initiation. Among these, leptin appears to provide an important permissive signal linking energy availability with activation of the hypothalamic–pituitary–gonadal axis. Insulin and hyperinsulinemia may further influence pubertal timing through effects on hypothalamic kisspeptin signaling and ovarian and adrenal androgen production. Additional mechanisms involving sirtuin-mediated epigenetic regulation of *Kiss1*, the ghrelin–LEAP2 system, and the gut microbiota have also been proposed. Environmental endocrine-disrupting chemicals, including bisphenol A and phthalates, may provide additional links between adiposity and puberty. Clarifying these links is clinically important for monitoring pubertal progression and cardiometabolic risk in children with obesity, and for informing prevention and early intervention aimed at reducing long-term adverse outcome.

## 1. Introduction

Childhood and adolescent overweight and obesity represent significant and escalating global health challenges. A comprehensive systematic review and meta-analysis of population-based studies from 154 countries or regions, with data collected between 2000 and 2023, found a pooled obesity prevalence of 8.5% among children and adolescents, and a pooled prevalence of excess weight (overweight or obesity) of 22.2%. The analysis also identified a higher prevalence of obesity in more recent periods, indicating a persistent increase in pediatric obesity [1]. In addition, a global pooled analysis of measured height and weight data from 200 countries and territories showed that the combined burden of thinness and obesity among school-aged children and adolescents increased in most countries between 1990 and 2022, with rising obesity as the primary driver of this trend [2].

Pubertal timing serves as a clinically significant developmental marker associated with various long-term health trajectories. Earlier onset of puberty has been linked to adverse cardiometabolic outcomes, including obesity, diabetes, and cardiovascular disease, as well as psychosocial and mental health risks such as behavioral dysfunction and depression, with some effects persisting into adulthood [3]. Systematic reviews further demonstrate that hormonal changes during puberty, particularly changes in testosterone and estradiol, are associated with specific mental health problems in children and adolescents [4]. Collectively, these findings highlight the importance of monitoring pubertal timing and its hormonal correlates in pediatric practice and emphasize the clinical relevance of secular trends toward earlier puberty at the population level [4].

Childhood overweight and obesity are consistently linked to earlier pubertal development, with the strongest evidence observed in girls. Meta-analyses indicate that overweight and obese girls have approximately twice the odds of experiencing earlier puberty compared to their normal-weight counterparts, with this association characterized as definite or strong across pooled data [4]. A large prospective cohort study employing sibling-matched analyses supports these findings, showing that overweight and obese girls reached pubertal milestones about five months earlier than normal-weight peers, and this association persisted after adjusting for shared familial confounders [5]. In boys, the evidence is less consistent. Although cohort data suggest earlier pubertal timing in overweight and obese boys compared to normal-weight peers, this association was attenuated and became non-significant in sibling-matched analyses, implying that shared familial factors as well as more complicated hormonal mechanisms may partially explain the observed association in males [5]. Some studies found no association between obesity and early puberty in girls but observed a significantly higher rate of delayed puberty in obese boys [6]. Meta-analytic estimates also suggest a trend toward earlier puberty in overweight and obese boys, but these findings do not reach statistical significance, with residual heterogeneity attributed to differences in definitions of pubertal markers, adiposity classification, and study design [5]. Beyond methodological heterogeneity, population characteristics including race and ethnicity also appear to modify the strength of the association in boys [7].

The underlying mechanisms are multifactorial, involving metabolic, hormonal, inflammatory, genetic, epigenetic, and environmental pathways that can affect hypothalamic–pituitary–gonadal (HPG) activation, adrenal androgen production, sex-steroid bioavailability, and subsequent clinical outcomes [8]. This work is conducted as a narrative review examining the relationship between childhood obesity and pubertal timing in girls. To inform the synthesis, a focused literature search was performed in **PubMed**, using relevant keywords and MeSH terms related to childhood obesity, adiposity, earlier puberty onset, secular trends in obesity and puberty onset. Articles were selected based on their relevance, methodological contribution, and impact on the current understanding of the metabolic and endocrine mechanisms linking excess adiposity with earlier pubertal onset.

This review explores the relationship between increased childhood adiposity and altered pubertal timing, secular trends in body weight, and the biological mechanisms through which excess adipose tissue may influence pubertal development.

## 2. Physiology of Normal Puberty

Puberty is a progressive developmental stage characterized by the establishment of reproductive capability, significant physical growth including the completion of skeletal lengthening, and concurrent psychological and behavioral maturation, collectively resulting in the adult phenotype [9]. The primary biological function of puberty is the activation of the HPG axis, enabling fertility, accompanied by coordinated somatic and psychological adaptations that prepare individuals for adult life [9]. The biological onset of puberty occurs in the brain, where alterations in stimulatory and inhibitory signals to gonadotropin-releasing hormone (GnRH) neurons modify their activity [9]. This process reinitiates the rhythmic release of pituitary gonadotropins after a period of functional quiescence of the HPG axis during childhood [9,10]. The HPG axis is not newly established at puberty; it is active in utero, reactivates during a transient neonatal phase known as mini puberty, and then remains relatively inactive until the onset of puberty [10].

Observational data indicate that the average age of pubertal onset has decreased by approximately twelve to eighteen months over the past two decades, prompting the reconsideration of the lower age limit for normal puberty, although consensus thresholds remain unchanged [11].

### 2.1. Neuroendocrine Regulation of Puberty

Within the hypothalamic network, KNDy neurons in the arcuate nucleus function as the principal pulse generator. These neurons coordinate GnRH neuron activity through the co-secretion of kisspeptin, neurokinin B, and dynorphin, which act on cell surface receptors to initiate each GnRH pulse [12]. Pubertal reactivation of pulsatile GnRH secretion stimulates anterior pituitary gonadotrope cells to release luteinizing hormone (LH) and follicle-stimulating hormone (FSH), which in turn act on the testes and ovaries to promote gametogenesis and sex-steroid production [12]. The identification of inactivating and activating mutations in the kisspeptin pathway, inactivating mutations in TACR3, and genetic findings implicating MKRN3 as a hypothalamic repressor of the gonadal axis, along with evidence of a developmental switch from inhibitory to excitatory hypothalamic input, have transformed pubertal physiology from a descriptive process to a partially mapped molecular framework [11,12].

### 2.2. Pulsatile GnRH and the Pituitary Response

Following hypothalamic reactivation, the critical determinant for maturation of the reproductive axis is the mode of GnRH delivery to the anterior pituitary. Intermittent exposure of the gonadotrope to GnRH (a decapeptide) sustains physiological secretion of LH and FSH in humans [13,14,15]. This principle is reflected in endocrine therapeutics. Continuous stimulation of the GnRH receptor by long-acting agonists initially induces a flare, followed by receptor down-regulation and suppression of gonadotropin secretion, a mechanism utilized in the treatment of precocious puberty [14]. In contrast, physiological pulsatile GnRH delivery restores gonadotropin secretion and reproductive function in hypogonadotropic states [14,15]. In healthy children, the earliest hormonal marker of impending puberty is a sleep-associated increase in pulsatile LH secretion. This is followed by a marked increase in LH pulse amplitude across pubertal stages, with the day–night difference in LH secretion only equalizing in late puberty [11]. As pubertal maturation advances, the evolving pattern of pulsatile gonadotropin output provides the sustained LH and FSH stimulation required by the gonad to drive steroidogenesis, as discussed in the following section [11,13,15].

### 2.3. LH, FSH, and the Sleep-Associated Signature of Early Puberty

Early in puberty, LH pulsatility demonstrates a distinct sleep-associated pattern. Polysomnography studies indicate that nocturnal LH pulses are most closely linked to slow-wave sleep and occur less frequently during wakefulness or rapid eye movement (REM) sleep. As puberty advances in girls, the frequency of sleep-associated LH pulses remains relatively stable at approximately one pulse every two hours. In contrast, waking LH pulse frequency gradually increases and, by late puberty, surpasses the sleep-associated frequency, resulting in a mature 24 h pattern of pulsatile secretion [16,17].

### 2.4. Steroidogenic Output During Gonadal Maturation

The pituitary signal described previously manifests physiologically as gonadal steroid output, with distinct cellular pathways in males and females. Gonadarche is the central, HPG axis-mediated activation of the ovaries or testes, leading to rising estradiol or testosterone levels [16]. In the testes, LH binds to its receptor on Leydig cells, activating cyclic adenosine monophosphate (cAMP)-dependent signaling, which increases cholesterol translocation into mitochondria. This process is mediated by a mitochondrial transport complex, in which the steroidogenic acute regulatory protein (StAR) enhances cholesterol transport, which represents the rate-limiting step in acute steroidogenesis. Sequential steroidogenic enzymes then convert cholesterol into testosterone. The adult Leydig cell population, which sustains androgen production, develops postnatally from stem cells and is regulated by LH-dependent signaling [18]. In the ovary, estradiol synthesis occurs through a two-cell, two-gonadotropin mechanism. LH stimulates thecal cells to synthesize androgens, which diffuse into the adjacent granulosa layer. Aromatase, under FSH regulation, converts these androgens into estradiol. Granulosa cells are the principal ovarian site of aromatase expression, and FSH regulates CYP19A1 via cAMP-protein kinase A (PKA) signaling. The increase in FSH-dependent granulosa cell activity provides the ovarian mechanism for converting thecal androgens into estradiol [19].

### 2.5. Adrenarche as a Parallel and Distinct Developmental Process

Adrenarche is the maturation of the adrenal zona reticularis, resulting in increased adrenal androgens (DHEA, DHEA-S) and the appearance of pubic or axillary hair, body odor, and mild acne [11,20]. Adrenarche occurs concurrently with, but independently from, the reactivation of the HPG axis. It is characterized by a maturational increase in adrenal C19 androgen secretion, reflecting functional differentiation of the zona reticularis [20,21]. Biochemically, adrenarche is most accurately indicated by increased dehydroepiandrosterone sulfate, a steroid with high concentrations, a long half-life, and minimal diurnal variation, providing a reliable marker of zona reticularis maturation [20]. The developing zona reticularis exhibits a unique enzymatic profile, including low 3β-hydroxysteroid dehydrogenase type 2 and elevated cytochrome b5A, sulfotransferase 2A1, and 17β-hydroxysteroid dehydrogenase type 5. Additionally, 11-ketotestosterone has recently been identified as a bioactive adrenarchal androgen that significantly contributes to circulating androgenic activity [21]. Clinically, adrenarche presents as pubarche, which includes the development of pubic and axillary hair, adult-type apocrine body odor, oily skin and hair, and mild acne. These features may precede or coincide with gonadarche, as adrenal androgen production is regulated independently of the HPG axis [20,21]. Premature adrenarche is defined if occurs in girls at age younger than 8 years and in boys younger than 9 years [11,22]. Notably, adrenarche occurs in individuals with hypogonadotropic hypogonadism and those with primary gonadal failure, indicating a clinical proof that its initiation is independent of gonadotropin stimulation [20].

### 2.6. Progression of Secondary Sexual Characteristics in Clinical Assessment

The Marshall–Tanner five-stage classification, which grades breast, genital, and pubic hair development from prepubertal stage 1 to fully mature stage 5, remains the standard framework for assessing the visible sequence of normal puberty in clinical practice [22]. In girls, the first externally visible sign is typically thelarche, assessed as palpable breast budding, while in boys the corresponding clinical landmark is testicular enlargement [22,23]. Pubic hair development reflects adrenal androgen activity rather than gonadarche alone; therefore, pubarche should be interpreted separately from thelarche. However, breast development is generally the initial clinical sign of pubertal initiation in girls [22]. In boys, the onset of puberty is more precisely defined by an increase in testicular volume to 4 mL or greater, accompanied by scrotal enlargement and changes in scrotal skin texture. This early increase in testicular volume is primarily due to FSH-stimulated Sertoli cell proliferation, whereas later testicular maturation reflects increasing intratesticular testosterone levels, Sertoli cell maturation, and progression of spermatogenesis [24]. Subsequent visible male milestones include penile and scrotal enlargement, progression of pubic and axillary hair, voice change, and eventually facial hair. Circulating testosterone is usually low or undetectable during Tanner stages 1–2, rises markedly during stages 3–4, and reaches adult male concentrations by Tanner stage 4 [23,24]. The onset of clinical pubertal development before 8 years of age in girls and before 9 years of age in boys, driven by premature activation of the HPG axis refers to central precocious puberty. Peripheral precocious puberty, also called gonadotropin-independent precocious puberty is distinct from central precocious puberty occurring again before the age of 8 years in girls and 9 years in boys, and is caused by excess production of sex steroids from peripheral sources. Early puberty describes pubertal body characteristics that occur within the normal physiological pathway but earlier than average, typically between 8 and 9 years in girls, without meeting criteria for precocious puberty [10,22,23].

### 2.7. Age Ranges and Sex Differences in Pubertal Progression

Recent population-based data demonstrate that pubertal milestones occur within a broad normal range and that puberty is a multi-year process rather than a single event [23]. In the Puberty Cohort of the Danish National Birth Cohort, which included 14,759 children, the mean age at menarche was 13.0 years (95% CI 13.0–13.1), and Tanner Breast Stage 5 was reached at 15.8 years (95% CI 15.7–15.9). Among boys, voice break occurred at 13.1 years (95% CI 13.0–13.1), first ejaculation at 13.4 years (95% CI 13.3–13.4), and Tanner Genital Stage 5 at 15.6 years (95% CI 15.5–15.6) [23]. These data indicate that, on average, girls attain mid-pubertal milestones slightly earlier than boys do, although the overall duration of pubertal progression is similar between the sexes [23]. A recent study by Mohsenipour et al., found no association between obesity and early puberty in girls, but observed a significantly higher rate of delayed puberty in obese boys [6]. Variation in pubertal timing in both obese boys and girls is influenced by genetic and epigenetic factors. Human genetic studies suggest that approximately 50–80% of the variation in pubertal onset is genetically determined with some sex differences [25]. Genetic research has identified common variants in both sexes associated with earlier pubertal timing and rarer variants of genes such as *MKRN3* and *DLK1* implicated not only in precocious but also in early puberty in both sexes [25]. Studies showed that higher kisspeptin levels and a greater hypothalamic kisspeptin activity as puberty approaches. Excess adiposity amplifies this pathway through increased aromatase activity, higher estradiol exposure, and enhanced leptin-mediated stimulation of Kiss1 neurons [23,24,25]. Furthermore, overweight and obese girls have earlier maturation of hypothalamic kisspeptin neurons, stronger estrogen-mediated up-regulation of kisspeptin expression, and distinct metabolic and adiposity-related cues such as leptin, insulin and adipokines that enhance kisspeptin signaling [11,12,23]. On the contrary, in obese boys, mechanisms may differ and the initial surge of testosterone in the beginning of puberty is plausibly suppressed by increased aromatization, leptin excess, and inflammatory inhibition of GnRH pulsatility [6,23,24,25]. In addition, kisspeptin neurons respond differently to leptin and insulin in females versus males, with female circuits showing greater sensitivity to metabolic cues that lower the threshold for HPG axis activation [25]. Finally, epigenetic regulation through kisspeptin is more permissive in girls under conditions of energy excess, allowing increased histone acetylation and earlier activation of pubertal genes, whereas boys exhibit a more resistant or inhibitory epigenetic profile [23]. Together, these mechanisms explain why obesity consistently accelerates puberty in girls but produces delayed or blunted pubertal progression in boys.

## 3. Childhood Obesity

### 3.1. Definition and Pathophysiology of Obesity

Obesity is a result of chronic positive energy balance. Excess energy is stored in the form of adipose tissue [26]. The degree of excess weight is commonly measured using the Body Mass Index (BMI) scale. Childhood obesity is defined by the World Health Organization (WHO) as abnormal or excessive fat accumulation that presents a risk to health. Because children’s body composition changes with age, WHO uses age- and sex-specific growth references rather than fixed BMI cutoffs. For children under 5 years, obesity is defined as weight-for-height more than 3 standard deviations above the WHO Child Growth Standards median. For children aged 5–19 years, obesity is defined as BMI-for-age more than 2 standard deviations above the WHO Growth Reference median [26]. Adipose tissue consists of many adipocytes that have an extensive involvement in the management of metabolism. Amongst the most notable are its effects on appetite regulation, insulin sensitivity and to a lesser extent the reproductive function. Additionally, adipose tissue serves as triacylglycerol storage, participates in inflammation and displays endocrine functions. Immune dysfunction in the form of low-grade chronic inflammation is often linked to obesity. Along with increased insulin resistance (IR), both are considered predisposing factors for multiple health issues and may directly or indirectly influence pubertal development. Obesity-associated inflammation is often driven by pro-inflammatory macrophages and the cytokines that they secrete [27]. Adipocytes secrete signaling molecules—adipokines, which have an important function in inflammation, regulation of appetite, satiety and body fat content [27]. TNF-a, leptin, resistin, visfatin, IL-6 and adiponectin are amongst the most researched adipokines and are of great significance in the context of our review.

In individuals with higher BMI, adipose tissue becomes more pro-inflammatory and higher amounts of leptin and resistin are secreted, which is correlated to an increase in IR [26,27]. Puberty alone is found to be associated with an elevation in IR; therefore, obesity is likely to exacerbate this effect, leading to increased risks of cardiometabolic diseases and type 2 diabetes [28].

### 3.2. Adipose Tissue Biology

Adipose tissue is divided into three different types, white adipose tissue (WAT), brown adipose tissue (BAT) and beige adipose tissue (BeAT). The primary function of WAT is to store excess energy in the form of large unilocular lipid droplets consisting of triglycerides. Later, when the stored energy is required by the body, it is released from adipocytes in a form of free fatty acids (FFAs) [29]. Adipose tissue is a major site of extragonadal aromatase activity, and its enzymatic capacity increases proportionally with fat mass. Aromatase (CYP19A1) converts circulating androgens—primarily androstenedione and testosterone—into estrone and estradiol, generating a continuous source of peripheral estrogens independent of ovarian activation. In children with obesity, the expanded adipose compartment produces higher baseline estrogen levels, which can stimulate breast tissue even when the HPG axis remains quiescent. This peripheral estrogen exposure may lead to earlier or more prominent thelarche, sometimes presenting as isolated premature thelarche. Beyond estrogen production, adipose tissue also expresses pro-inflammatory cytokines and adipokines that can further modulate aromatase expression, reinforcing the link between excess adiposity and early breast development [29].

### 3.3. Obesity and Insulin Resistance

It is well established that both obesity and puberty are associated with elevated insulin resistance (IR), altering normal insulin function [28,29]. Insulin has the ability to cross the blood–brain barrier and act on the insulin receptors within both GnRH-secreting neurons and kisspeptin neurons [30]. Therefore, elevated levels of insulin are thought to influence the onset of puberty through metabolic signaling [31]. Mice of both genders lacking Kiss1 insulin receptors showed a significant delay in sexual maturation [32]. This may indicate the role that insulin plays in puberty. A different study looked at mice with high insulin levels and found a correlation with earlier pubertal onset, however there was no significant effect of insulin on the progression of the pubertal changes [30]. Hence, hyperinsulinaemic state without co-existent obesity is insufficient to fully replicate obesity-associated early pubertal changes. Furthermore, a theory was proposed, that there might be a positive correlation between insulin levels and kisspeptin synthesis, suggestive of insulin being a contributing factor towards activation of the HPG system. However, this area requires further research to determine the presence of significant associations, as evidence remains limited [30,32].

### 3.4. Causes of Obesity: Multifactorial Framework

Due to its pathophysiological complexity and multifactorial nature, it is challenging to underline a specific singular contributor having the most impact on the development and progression of excessive weight gain. Amongst most notable are biological, genetic, developmental, environmental and psychosocial factors [33].

#### 3.4.1. Biological Factors Influencing Obesity

Orexigenic hormones such as ghrelin and anorexigenic hormones from pancreas, gut and adipose tissue play a critical role in appetite regulation. They do so through various signaling pathways within the arcuate nucleus (ARC) of the hypothalamus. Disruption in this system may predispose to obesogenic behavior, hence resulting in a consequent weight gain. A wide variety of factors have an influence on such hormones, however one of the more interesting appears to be abnormal sleep–wake cycle and elevated stress has been associated with increased secretion of hunger-stimulating hormone ghrelin [33].

#### 3.4.2. Enteric Microbiome and Obesity

The human gut microbiome plays a major role in energy and metabolism regulation [33]. Dysbiosis is frequently observed along with obesity and may lead to increased energy harvest in certain cases. Alterations of enteric bacteria may also be linked to precocious puberty, due to their proposed regulatory effects on LH, FSH and insulin. However, this field remains poorly understood and requires further research [34].

The gut microbiota is influenced by a multitude of various factors involving antibiotic use, genetics, diet, lifestyle and early development of the child. Reduced microbiome diversity has been linked with low-grade inflammation and alterations in the production of short-chain fatty acids (SCFAs), both being associated with the pathophysiology of obesity [33]. Inflammation in such cases is caused by increased gut permeability, allowing elevated translocation of lipopolysaccharides from Gram-negative bacteria into circulation. This process is known as metabolic endotoxemia and may exacerbate IR.

Altered gut microbiome is also associated with disrupted feeding behavior and subsequent hyperphagia. Bacteria do so by influencing the production of gut-derived hormones such as GLP-1, PYY and Ghrelin [35].

#### 3.4.3. Role of Genetics and Epigenetics in Obesity

Upwards of 900 genetic loci have been linked to obesity. They are involved in basal metabolic rate regulation, leptin sensitivity, and many more. Studies on monozygotic twins born to obese parents have shown a 40–75% greater risk of obesity development [36].

Generally, genetic causes present as either monogenic or polygenic [37]. Monogenic obesity is often driven by mutations within *leptin*/*POMC* genes and is very rare. Polygenic obesity, on the other hand, is influenced by a combination of multiple genetic factors, hence a higher prevalence. Such genetic background contributes towards accelerated weight gain in an obesogenic environment [37].

Epigenetic changes occur within human cells altering the expression of certain genes when exposed to an obesity-promoting environment. This process, in combination with polygenic predisposition, may significantly predispose to a more rapid and severe weight gain. Epigenetic changes can be induced by other factors, such as maternal diet throughout prenatal and postnatal periods, psychosocial stressors and fetal malnutrition. Moreover, maternal obesity has been associated with a threefold increase in the risk of childhood obesity. Maternal stress, exposure to air pollutants, assisted reproduction, smoking habits and fathers’ excessive preconception weight are linked to an increased risk of obesity amongst offspring [33,36].

#### 3.4.4. Endocrine Disrupting Chemicals—Environmental and Lifestyle Factors

Familial and environmental factors significantly influence dietary behaviors in children. Parental obesity is a strong predictor of a child’s BMI. Similar calorie-dense diets along with genetic predispositions often lead to a higher BMI among children in such families. Additionally, the younger generation spends more time behind the screen and are more likely to be exposed to targeted food advertising [36].

The exposome—the totality of environmental exposures across the life course—provides a useful framework for understanding how external factors shape both adiposity and pubertal timing. Endocrine-disrupting chemicals (EDCs) such as bisphenol A (BPA), phthalates, parabens, PFAS, and flame retardants exert heterogeneous estrogenic, antiandrogenic, metabolic, and epigenetic effects, but human evidence remains inconsistent and far from definitive. While animal and in vitro studies demonstrate that these compounds can alter adipogenesis, insulin signaling, hypothalamic development, and steroidogenesis, human studies are limited by exposure misclassification, mixtures, non-monotonic dose–response relationships, confounding (e.g., diet, socioeconomic status), and potential reverse causation—for example, higher adiposity may increase storage of lipophilic chemicals, making exposure appear causally related [36,37]. Phthalates and bisphenol A (BPA) are chemicals commonly found in everyday use plastics and come in contact with food. BPA competes with endogenous estradiol for its receptors due to its estrogenic nature. Estrogen-mimicking properties of BPA may link its association to early onset of puberty, especially in girls [38].

At the hypothalamic level, EDCs disrupt the Kiss1–GnRH neuronal network by modifying epigenetic marks such as DNA methylation and histone acetylation, thereby shifting chromatin accessibility at *Kiss1*, *Tac3*, and *GnRH* regulatory loci. Several compounds—including bisphenols, phthalates, and PFAS—alter SIRT1 activity, NAD^+^ availability, and histone deacetylation, creating an imbalance between repressive (H3K27me3) and activating (H3K9ac, H3K14ac) histone marks. Through these mechanisms, EDCs can prematurely relieve repression of *Kiss1* or, conversely, enhance silencing depending on receptor affinity and timing of exposure. In parallel, EDC-induced metabolic changes—adipogenesis, leptin elevation, insulin resistance, and altered SCFA signaling—feed back to the hypothalamus and further modulate GnRH pulsatility. The combined endocrine, metabolic, and epigenetic disruption produces non-monotonic, sex-specific shifts in pubertal onset, reflecting the sensitivity of the HPG axis to environmental chemical cues during critical developmental windows [38].

Beyond chemical exposures, lifestyle and social environmental factors interact biologically with both obesity risk and pubertal timing. Diets high in ultra-processed foods, added sugars, and obesogenic additives promote positive energy balance, insulin resistance, and increased leptin, accelerating hypothalamic maturation [37,38]. Physical inactivity and excessive screen time reduce energy expenditure and are associated with altered melatonin rhythms and increased adiposity, both of which may influence pubertal onset. Psychosocial stress—including family conflict, adverse childhood experiences, and chronic socioeconomic disadvantage—activates the HPA axis, increases cortisol, and may hasten maturation through stress-related pathways described in evolutionary biology models. Short sleep duration disrupts circadian regulation of appetite hormones and may indirectly affect pubertal timing through increased adiposity and altered melatonin signaling. Together, these environmental and lifestyle exposures form an interconnected network influencing metabolic status and neuroendocrine maturation, highlighting the need for integrative, multi-level approaches when interpreting associations between obesity and early puberty in girls [36,37].

## 4. Molecular Mechanisms Linking Obesity to Early Puberty

### 4.1. Leptin

Excess adipose tissue has been associated with higher leptin production; however, leptin should be regarded primarily as a permissive signal of adequate energy availability rather than as a simple direct trigger of puberty [39,40,41]. Interactions between leptin and kisspeptin systems result in HPG axis activation through KNDy neurons located within the ARC of the hypothalamus [40,41]. Kisspeptin–Kiss1R signaling is essential for the initiation of GnRH release from the hypothalamus. Females possess higher concentrations of kisspeptin than males, in part explaining why girls are more affected by precocious puberty. However, this hypothesis is not universally established. Nevertheless, leptin increases Kiss levels through KNDy hypothalamic neurons, leading to GnRH secretion [41]. In addition, leptin influences GnRH secretion through inhibition of NPY and concurrent stimulation of POMC and α-MSH [42]. It also exerts its function by directly promoting LH and FSH secretion from gonadotrophs within the anterior pituitary gland [43]; however, its effects are minor. Furthermore, leptin receptor isoforms have been identified in human granulosa and theca cells and in rodent Leydig cells; however, the clinical significance of these findings regarding pubertal timing in children remains uncertain [44,45]. Additionally, leptin is indirectly associated with compromised testosterone production and spermatogenesis [41].

### 4.2. Insulin

Although hyperinsulinemia may modify pubertal phenotypes, current evidence does not support that insulin displays a role of direct initiator of central puberty [46]. In children with obesity, insulin resistance and the resultant hyperinsulinemia result in increased production of androgens by the ovaries and adrenal glands and causes reduction in hepatic sex hormone-binding globulin (SHBG) [41,43,46]. These hormonal events increase the levels of androgens and sex steroids which contribute substantially to pubertal development. Moreover, insulin stimulates adipocytes to further produce and secrete leptin [47], thereby influencing GnRH secretion through the above-discussed mechanisms. However, insulin may also promote directly the pulsatile release of GnRH through binding onto its cognate receptor in hypothalamus leading to increased *KISS1* expression [30]. It is worth mentioning that hyperinsulinemia in prepubertal girls with obesity results in acceleration of pubertal development secondary to the positive feedback loop exerted on the HPG axis, whereas prepubertal boys with obesity tend to show a delay in pubertal onset [47].

### 4.3. Sirtuins

Sirtuins are NAD^+^-dependent enzymes that regulate glucose, lipid, and amino acid metabolism, mitochondrial function, and cellular stress responses. In mammals, they play a central role in maintaining energy homeostasis in relation to nutritional status. Among them, Sirtuin 1 (SIRT1) is highly expressed in the hypothalamus and in several peripheral metabolic tissues [48]. Nutrient deprivation increases the intracellular NAD^+^/NADH ratio, leading to enhanced SIRT1 activity and expression across multiple organs [49,50]. Through this mechanism, SIRT1 acts as a molecular sensor of energy deficit and coordinates adaptive metabolic responses at both central and peripheral levels [51].

**Figure 1:**
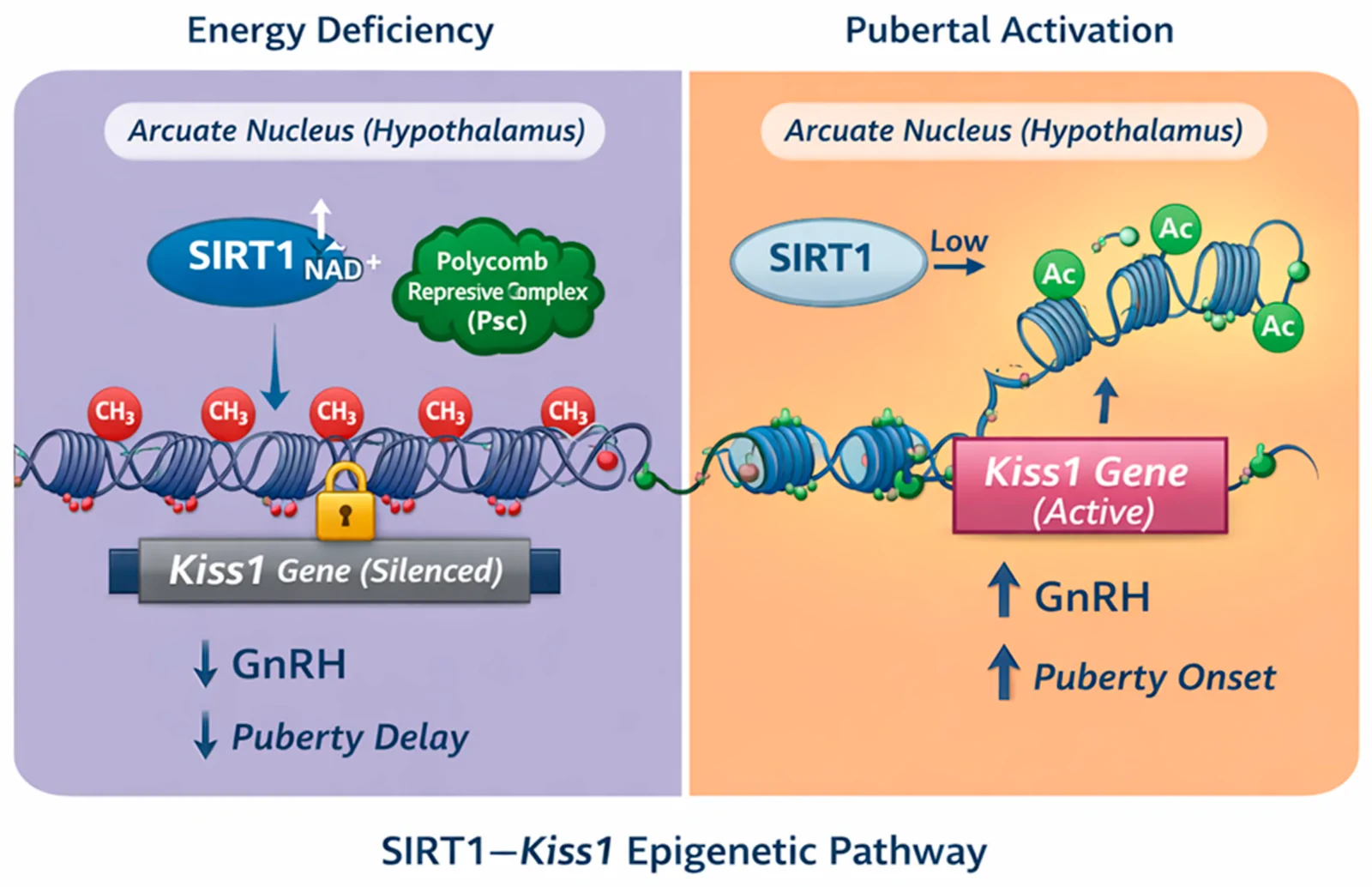
SIRT1-Kiss1 epigenetic pathway model. As puberty approaches, SIRT1 is progressively displaced from the Kiss1 locus, allowing chromatin relaxation and activation of Kiss1 transcription, which in turn stimulates GnRH release. Conversely, increased SIRT1 activity during energy deficit maintains chromatin in a repressed configuration, prolonging Kiss1 silencing and delaying pubertal initiation. (Polycomb Repressive Complex (Psc), methyl groups (CH_3_), Histone acetylation (Ac)). Figure 1 was designed using AI.

A key developmental function of SIRT1 involves the epigenetic regulation of the *Kiss1* gene, a critical upstream activator of GnRH secretion. In the arcuate nucleus, SIRT1 interacts with the Polycomb silencing complex (Psc), a major epigenetic repressor that maintains chromatin in a transcriptionally inactive state. Together, SIRT1 and Psc compact chromatin around the *Kiss1* promoter, suppressing its expression. As puberty approaches, SIRT1 is progressively displaced from the *Kiss1* locus, allowing chromatin relaxation and activation of *Kiss1* transcription, which in turn stimulates GnRH release. Conversely, increased SIRT1 activity during energy deficit maintains chromatin in a repressed configuration, prolonging *Kiss1* silencing and delaying pubertal initiation [52]. Experimental models demonstrate that early onset overnutrition reduces hypothalamic SIRT1 levels and accelerates Kiss1 expression, thereby advancing puberty, whereas undernutrition elevates SIRT1, prolongs *Kiss1* repression, and delays pubertal onset [53] (Figure 1).

Beyond its role in Kiss1 regulation, SIRT1 also modulates hypothalamic circuits governing appetite. Changes in appetite and metabolism during puberty may illustrate an indirect role of SIRT1 in puberty timing. SIRT1 regulates the activity of Agouti-related peptide (AgRP) and proopiomelanocortin (POMC) neurons, which produce orexigenic and anorexigenic neuropeptides, respectively. During fasting, increased SIRT1 activity enhances AgRP expression and promotes food intake. Inhibition of hypothalamic SIRT1 reduces AgRP and increases POMC expression, shifting the balance toward appetite suppression. Under conditions of energy sufficiency, reduced SIRT1 activity contributes to decreased AgRP signaling and lower food intake [54].

Emerging evidence in humans indicates that physical activity increases SIRT1 and SIRT3 expression, whereas sedentary behavior which is common in childhood obesity is associated with reduced sirtuin activity. Lower SIRT1 levels may diminish repression of *Kiss1*, contributing to earlier pubertal onset in obese girls [55]. These mechanisms provide a plausible biological framework linking nutritional status, physical activity, and pubertal timing.

A limited number of studies have examined SIRT1 in pediatric populations, and results are inconclusive regarding its direct role in the secular trend toward earlier puberty. A recent study with a small sample size reported that serum SIRT1 concentrations in healthy children did not differ by sex, age, anthropometric measures, or pubertal stage. Interestingly, higher consumption of fruits, vegetables, and dairy products was associated with increased SIRT1 levels, suggesting potential dietary influences on sirtuin activity [56,57].

### 4.4. LEAP2

Liver-enriched antimicrobial peptide 2 (LEAP2), originally isolated from human serum in 2003 due to its structural similarity to innate antimicrobial peptides, has recently emerged as a key metabolic hormone with major implications for growth and pubertal regulation. Early work demonstrated that LEAP2 mRNA is predominantly expressed in the liver, with lower expression in the kidney, jejunum, duodenum, stomach, and heart, suggesting both hepatic and gastrointestinal contributions to circulating levels [58]. A major conceptual shift occurred with the discovery that LEAP2 is an endogenous antagonist of the growth hormone secretagogue receptor (GHSR), the canonical receptor for ghrelin. In elegant mechanistic studies, Ge et al. (2018) showed that LEAP2 completely suppresses ghrelin induced GHSR activation, thereby blocking ghrelin’s major physiological actions, including the stimulation of appetite, promotion of GH secretion, and maintenance of glucose homeostasis during caloric restriction [59]. Conversely, inhibition of endogenous LEAP2 amplifies ghrelin signaling, underscoring the dynamic antagonistic balance between these two peptides [59].

Human studies further highlight LEAP2 as a possible nutritional and body mass-dependent regulator. LEAP2 concentrations rise with feeding and obesity and fall during fasting, mirroring the opposite pattern of ghrelin. In both humans and mice, LEAP2 levels increase with higher BMI and caloric intake, positioning LEAP2 as a possible counter regulatory hormone that restrains ghrelin’s orexigenic and GH releasing effects when energy stores are abundant [60]. This reciprocal ghrelin–LEAP2 relationship is particularly relevant during childhood, when changes in body composition and energy balance accompany the transition from prepuberty to puberty.

Ghrelin itself is a pleiotropic metabolic and neuroendocrine regulator. Beyond its well-established role in GH release—acting synergistically with GHRH—ghrelin promotes positive energy balance by increasing appetite and reducing fat utilization [61]. Importantly, ghrelin also exerts inhibitory effects on the reproductive axis. Experimental studies demonstrate that ghrelin suppresses luteinizing hormone secretion and delays pubertal onset, functioning as a metabolic brake on reproductive maturation during periods of energy insufficiency [62]. In humans, circulating ghrelin levels decline with advancing pubertal stage and show negative correlations with BMI and IGF1, consistent with the concept that reduced ghrelin signaling removes inhibitory tone on the hypothalamic–pituitary–gonadal axis as puberty progresses [63].

Taken together, these findings support a model in which the ghrelin–LEAP2 axis acts as a metabolic gatekeeper, integrating nutritional status with growth and pubertal timing. During prepuberty and puberty, shifts in BMI and energy balance may modulate the relative activities of ghrelin and LEAP2, influencing GH secretion, appetite regulation, and the timing of reproductive maturation. Such models pave the way for additional research on LEAP2 and its relevance to obesity-related early puberty, and metabolic influences on pubertal development.

### 4.5. Gut Microbiota

The gut–brain axis contributes to childhood obesity via bidirectional endocrine, metabolic and neural mechanisms [64]. Indeed, a variety of microorganisms, termed as the “microbiota”, influence the levels of several appetite-controlling hormones, including leptin, ghrelin and glucagon-like peptide (GLP-1); however, the specific mechanisms underlying the molecular communication between the gut microbiota and the neuroendocrine system remain to be elucidated [41]. For instance, although short-chain fatty acids (SCFAs) are well-recognized bacterial metabolites that influence substantially the activity of gut–brain axis, a small number of studies showed increased levels of SCFAs have been associated with the development of central precocious puberty through up-regulation of the leptin gene [65,66]. On the other hand, Wang and collaborators found an association between the reduced levels of SCFAs and early puberty in children with obesity [67]. Further studies are needed to elucidate the association between gut microbiota and obesity-associated early puberty. Table 1 summarizes the experimental and clinical evidence supporting the metabolic and pubertal regulatory pathways studies used in this review.

## 5. Long-Term Cardiometabolic Outcomes of Pubertal Obesity

Excess body weight is a risk factor seen in multiple pathologies such as type 2 diabetes mellitus (T2DM), non-alcoholic fatty liver disease, non-alcoholic steatohepatitis, cardiovascular disease (CVD) and several kinds of malignancies [29]. A Swedish study of 37,672 men looked at how BMI change during puberty impacted risks of death from cardiovascular conditions later in life. Results showed that participants who became obese during puberty had a 2.39-fold increased risk in cardiovascular death in adulthood. Boys who were overweight throughout both childhood and puberty had a 1.85-fold increase. Notably, childhood obesity which normalized during puberty had no significant effect on cardiovascular mortality [68]. These results confirmed the conclusions of a systematic review and meta-analysis published by Prentice and Viner who found that earlier pubertal onset was predictive of higher adult BMI and increased risk of obesity, partially independently of childhood BMI [69]. Furthermore, an earlier development of puberty has been associated with increased risk of other cardiometabolic risk factors and cardiovascular mortality [69].

In addition to long-term adverse cardiometabolic outcomes, it is worth mentioning that adolescents should be regularly followed up to monitor the progression of pubertal development, since pubertal obesity has been associated with early or delayed puberty. Furthermore, puberty is characterized by a startlingly high dropout rate among patients with obesity; therefore, it is of paramount importance to apply strategies to ensure appropriate monitoring of the physiological progression of puberty [70].

## 6. Concluding Remarks and Future Perspectives

**Figure 2:**
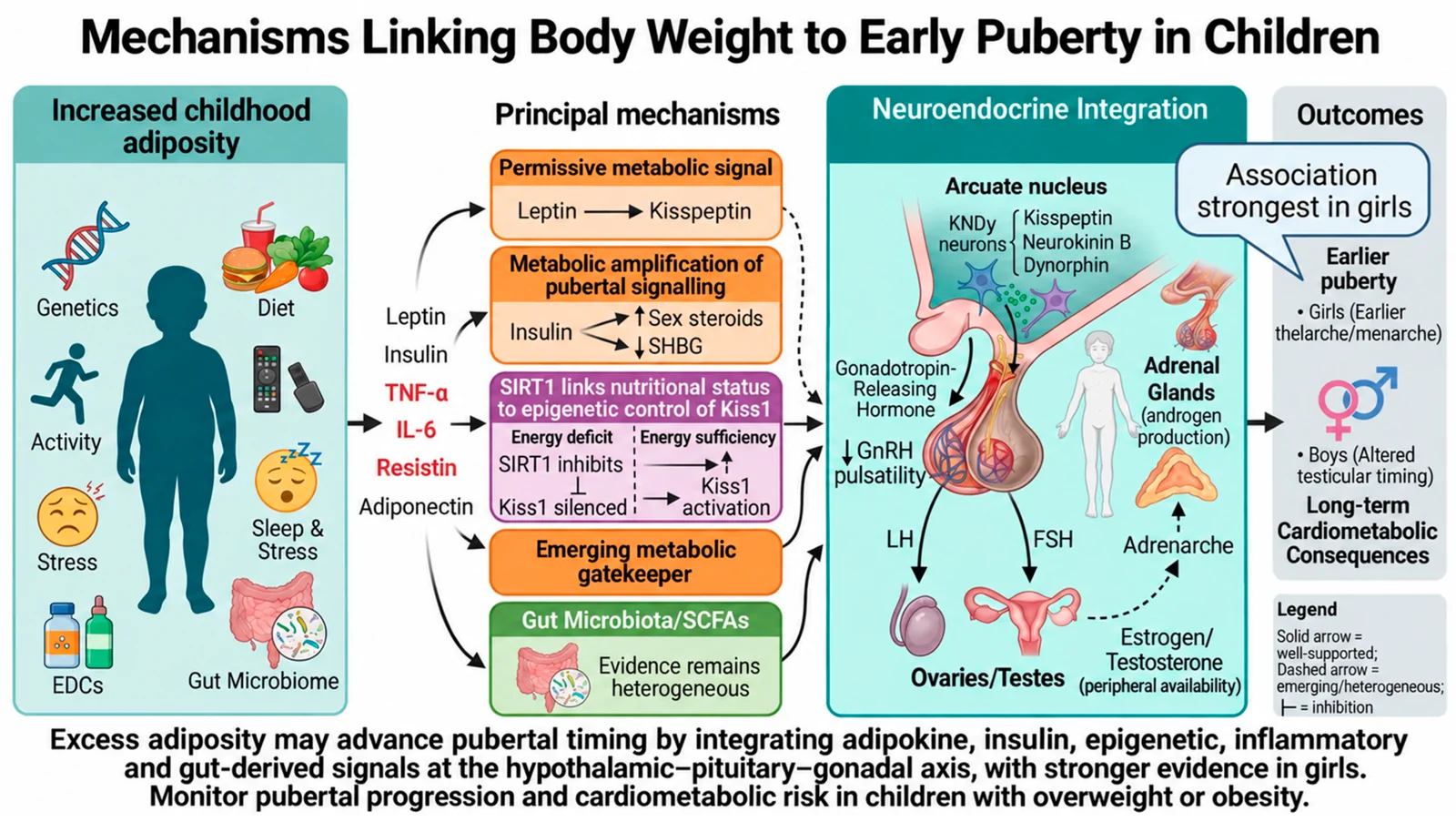
Mechanisms linking increased body weight to early development of puberty through central HPG-axis activation, adrenal androgen production, or peripheral sex-steroid availability. Solid arrows show better-supported pathways, whereas dashed arrows indicate emerging, heterogeneous, or predominantly experimental pathways. Arrows represent a conceptual framework rather than proof of causality. Earlier pubertal timing or isolated signs, especially in girls, do not pose a diagnosis of central precocious puberty. SCFAs, short-chain fatty acids; SHBG, sex hormone-binding globulin. Figure 2 was designed using AI.

Although several factors and mechanisms have been proposed to explain the association between childhood obesity and early pubertal onset, including leptin, insulin, sirtuins, LEAP2 and gut microbiota (Figure 2), their evidential support is substantially different. Leptin is considered as a permissive metabolic signal, whereas insulin may alter androgen production by adrenal glands and ovaries, and sex-steroid availability. It is worth mentioning that the central activation of HPG axis by either hormone remains incompletely demonstrated. Evidence regarding the role of SIRT1, LEAP2 and gut microbiota is still experimental and requires validation in longitudinal pediatric studies.

In addition to the role of the above-discussed signals, there are some limitations regarding the establishment of a clear causal relationship between increased body weight in childhood and puberty. Most human studies are observational, and pubertal development itself alters body composition, insulin sensitivity, fat distribution, sex-steroid concentrations, and subsequent BMI trajectories. Therefore, these associations are susceptible to reverse causality and residual confounding. Moreover, excess adiposity in girls with obesity may make clinical assessment of glandular breast tissue more difficult and may introduce misclassification in epidemiological studies. Furthermore, the definition of childhood overweight and obesity takes into account that BMI does not consider the distribution of adipose tissue, especially in adolescents with growth spurts. Finally, the number of factors contributing to obesity in children is ever-increasing, including lifestyle patterns, ethnicity traits and environmental factors. Undoubtedly, a novel field of –omics technologies, termed as “exposomics” might further delineate the influence of the exposure of children to environmental factors that both contribute to obesity and pubertal onset.

## Figures and Tables

**Table 1:** Evidence mapping of SIRT1, Ghrelin–LEAP2, and gut microbiota studies across animal, in vitro, and human research.

Evidence Type	Reference	Key Note/Contribution
**Animal Studies**	Satoh et al., 2010 [50]	SIRT1 activates hypothalamic nuclei to mediate adaptive response to diet restriction.
	Vazquez et al., 2005 [52]	SIRT1 regulates ovulatory hormonal trigger under energy deficit.
	Vazquez et al., 2018 [53]	SIRT1 epigenetically represses *Kiss1*, altering pubertal timing in obesity.
	Sasaki et al., 2010 [54]	Hypothalamic SIRT1 induction suppresses feeding via AgRP neurons.
	Ge et al., 2018 [59]	LEAP2 identified as endogenous antagonist of ghrelin receptor; includes mouse data.
	Mani et al., 2019 [60]	LEAP2 changes with body mass and food intake in mice (and humans).
	Wang et al., 2022 [67]	SCFAs modulate HPGA and reverse obesity-induced precocious puberty in female rats.
**In vitro/Model studies**	Cohen et al., 2004 [49]	Calorie restriction induces SIRT1 expression in mammalian cells.
	Krause et al., 2003 [58]	Isolation and biochemical characterization of LEAP2 peptide.
**Human Studies**	Fedorczak et al., 2023 [55]	SIRT1 involvement in GH/IGF-1 signaling and growth processes in children.
	Pacifici et al., 2019 [56]	Physical activity and SIRT1/SIRT3 activation improve glucose homeostasis.
	Fedorczak et al., 2024 [57]	Serum SIRT1 levels vary by sex, age, puberty stage, body weight, and diet.
	Mani et al., 2019 [60]	LEAP2 changes with body mass and food intake in humans (and mice).
	Dong et al., 2020 [66]	Gut microbiota composition associated with idiopathic CPP in girls.

## Data Availability

No new data were created or analyzed in this study. Data sharing is not applicable to this article.
